# Hypertension Persisting after Pre-Eclampsia: A Prospective Cohort Study at Mulago Hospital, Uganda 

**DOI:** 10.1371/journal.pone.0085273

**Published:** 2013-12-31

**Authors:** Annettee Nakimuli, Alison M. Elliott, Pontiano Kaleebu, Ashley Moffett, Florence Mirembe

**Affiliations:** 1 Department of Obstetrics and Gynaecology, Makerere University College of Health Sciences, Kampala, Uganda; 2 Medical Research Council/Uganda Virus Research Institute, Uganda Research Unit on AIDS, Entebbe, Uganda and London School of Hygiene and Tropical Medicine, London, United Kingdom; 3 Medical Research Council/Uganda Virus Research Institute, Uganda Research Unit on AIDS, Entebbe, Uganda; 4 Department of Pathology, University of Cambridge, Cambridge, United Kingdom and Centre for Trophoblast Research, Cambridge, United Kingdom; University of Tennessee Health Science Center, United States of America

## Abstract

**Background:**

Pre-eclampsia/eclampsia usually resolves after delivery but sometimes hypertension persists and cardiovascular disease develops later. Our objective was to determine the incidence and maternal socio-demographic and obstetric risk factors for persistence of hypertension in women with pre-eclampsia/eclampsia.

**Methods:**

This was a prospective cohort study conducted from July 2009 to June 2011 at Mulago Hospital labour ward and postnatal clinics. We followed up 188 women admitted with pre-eclampsia/eclampsia until 3 months after delivery. Data was collected using interviewer-administered questionnaires, examination of participants and review of medical records. Stata (version12) software was used for data analysis. Univariable analysis was used to compute the relative risk of persistent hypertension at the 95% confidence level. This was followed by multivariable logistic regression analysis to determine factors independently associated with persistence of hypertension.

**Results:**

64 (34%) out of the 188 women analysed had persistent hypertension three months after delivery. Maternal age, gestational age at delivery and parity were predictors of persistent hypertension.

**Conclusion:**

The proportion of women with pre-eclampsia/eclampsia at risk of persistent hypertension at three months after delivery was high, with nearly one of three mothers remaining hypertensive. Follow up of mothers who develop pre-eclampsia is important so that early diagnosis and management of chronic hypertension can be made to avoid long term morbidity and mortality.

## Introduction

****

Pre-eclampsia is a life threatening multi-system disorder of pregnancy [1,2] that is generally defined as new-onset hypertension of 140/90 mmHg or more and 24 hour proteinuria of 0.3g or more, occurring after 20 weeks of gestation [3,4]. Pre-eclampsia is a leading cause of maternal morbidity and mortality worldwide, complicating 6-8% of all pregnancies [5] and responsible for at least 9% of maternal deaths in Africa [2]. The disorder is also a major cause of perinatal morbidity and mortality globally, especially from preterm delivery [6-8]. Hypertension is a key feature of pre-eclampsia and generally resolves by 3 months after delivery but this is not always the case, leaving affected women with its associated risks [9]. For some women hypertension and other complications such as renal disease are due to a pre-existing clinical problem, but in others pre-eclampsia is the first indication that women might develop these disorders long after the pregnancy [10]. This situation could arise as a consequence of organ damage caused by pre-eclampsia or pre-eclampsia could be a risk factor of later disease [11] but the underlying pathogenesis is poorly understood. It is therefore important to follow up all pre-eclamptic women after delivery to enable early diagnosis of chronic hypertension and other complications because prompt treatment reduces severe morbidity and mortality. 

Although chronic hypertension is the major clinical problem to develop after pre-eclampsia [9,12], other diseases have been identified. These include ischaemic heart disease [13,14], stroke [15,16], renal disease [17] and Type 2 diabetes [18,19]. Previous studies have identified a number of predictive factors for later hypertension after pre-eclamptic pregnancies and the these include severe and/or early onset pre-eclampsia (before 34 weeks of gestation), recurrent pre-eclampsia and advanced maternal age [20-24]. The pathogenesis of pre-eclampsia is unclear but in the majority of cases there is associated failure of placentation, in particular the physiologic transformation of spiral arteries, leading to a stressed and under-perfused placenta [25,26]. A specialized type of immune cells called uterine natural killer cells are believed to be important in regulating trophoblast behavior during placentation via their Killer Immunoglobulin-like receptors (KIR) [27]. Indeed, certain combinations of maternal *KIR* genotypes and fetal *HLA-C* genes have been associated with pre-eclampsia among people of European descent [28]. It has been previously hypothesized that in response to the hypoxia that results from abnormal placentation the placenta releases some factors that lead to endothelial dysfunction and subsequent multisystem organ dysfunction that is characteristic of pre-eclampsia [29]. This organ dysfunction could lead to persistence of hypertension depending on the severity of the endothelial dysfunction, which could partly depend on the woman’s *KIR* genotype that may reflect natural killer cell activity. However, the association of maternal *KIR* genotype and resolution of pre-eclampsia has not been investigated before. Pre-eclampsia is a major cause of maternal death globally [2] and women of African origin are at a higher risk compared to other racial groups [30-34]. In Mulago Hospital cases with pre-eclampsia constitute at least 8% of admissions to the labour ward [24]. Very little is known about the long-term outcomes of pre-eclampsia in an African setting. We have therefore conducted a study to evaluate the incidence and factors associated with persistent hypertension in women admitted with pre-eclampsia/eclampsia at Mulago Hospital who were followed up for 3 months after delivery. 

## Methods

### Study Site and Population

The study was conducted at Mulago, Uganda’s national referral hospital, located in Kampala with a catchment area of Kampala District and the surrounding districts of Wakiso, Mukono and Mpigi. These surrounding districts are within a radius of 10 to 20 km from Mulago Hospital. Few obstetricians are available in the public hospitals in the districts and therefore women with complications such as pre-eclampsia are referred to Mulago Hospital where the obstetricians are and the health care services are free. Mulago Hospital occasionally receives referrals from other parts of the country. In addition to the referred women, local women routinely deliver in Mulago Hospital and there are 80-90 deliveries daily and more than 30,000 deliveries per year. This was a prospective cohort study conducted in the labour ward and postnatal clinic between July 2009 and June 2011 and was linked to a larger case-control study primarily designed to study the genetics of pre-eclampsia (Nakimuli, manuscript in preparation). The genes studied were *Killer Immunoglobulin-like receptor (KIR*) and *Human Leukocyte Antigen C (HLA-C*) and their role in development of pre-eclampsia in an African population was investigated. 

 Participants in the case-control study were women with pre-eclampsia or eclampsia consecutively recruited after admission to Mulago Hospital. Pre-eclampsia was defined as presence of hypertension and proteinuria occurring after 20 weeks of gestation. Hypertension was defined as a blood pressure (BP) measurement of 140/90 mmHg or more, measured at rest on more than one occasion at least 4 hours apart. Proteinuria was defined as urine protein measurement of +2 or more by dipstick on more than one occasion at least 4 hours apart. Severe pre-eclampsia was defined as presence of hypertension of 160/110 mmHg or more and proteinuria of 3+ or more at any time during management. Mild pre-eclampsia was defined as presence of hypertension of less than 160/110 mmHg and proteinuria of 2+ at any time during management. Eclampsia was diagnosed when a patient with pre-eclampsia had generalized tonic-clonic convulsions. All cases were at 20 weeks of gestation or more at the time the diagnosis of pre-eclampsia was made, assessed by weeks of amenorrhoea or ultrasound scan. 

 The majority of the women recruited to the larger genetic case-control study were included in the prospective follow up study apart from those who had no reliable residential address or phone contact and those living beyond a radius of 20 Km from Mulago Hospital due to difficulties in follow up. Women who missed reviews were traced by phone or midwives were sent to trace them personally to minimize loss to follow up. Women with prior chronic hypertension, renal disease before or during that pregnancy were also excluded because they already had the outcome of interest (persistent hypertension). These were self reported diagnoses and not backed my medical records. Some women reported using antihypertensive medication. Women with BP measurements of 140/90 mmHg or more before 20 weeks of gestation were also excluded as these are considered to have chronic hypertension. Participants were followed up for 3 months after delivery and persistent hypertension was diagnosed if by 3 months after delivery a woman had a blood pressure of 140/90 mmHg or more, or if antihypertensive medication was required to keep the BP below 140/90 mmHg. 

### Ethics statement

Approval to conduct the study was given by the Higher Degrees Research and Ethics Committee of Makerere University College of Health Sciences and the Uganda National Council for Science and Technology (UNCST). The participants gave written informed consent to participate in the study. Permission was sought to study minors (below 18 years of age) from the ethics committees. Minors signed assent forms while their next of kin or caretakers signed the consent forms. Withdrawal from the study never jeopardized health care and this was provided free to all women. 

### Data Collection

The women were interviewed and BP measurement taken by the same medical attendant (AN). Additional information was obtained from participants’ medical charts. The data collected included socio-demographic characteristics, ethnic group, obstetric, medical and present pregnancy history (gestational age at onset of pre-eclampsia and severity of pre-eclampsia). The Ethnic group was determined by asking women what their fathers’ languages were and this information was not used as a basis for recruitment in to the study. No biochemical data was collected as part of this study. Blood pressure measurements were done using manual mercury sphygmomanometers in a sitting position. Urine protein was measured using dipsticks. 5 mls of blood was taken from women and maternal genomic DNA was isolated using the QIAmp DNA Maxi Blood Kit (QIAGEN). KIR genotyping was based on the primers and methods described previously [28,35]. 

The sample size was estimated according to Kesley at al using OpenEpi version 2.3.1 software [36]. The incidence of persistent hypertension among all pre-eclamptics was 28% in an earlier study in the same study setting as ours [24]. The assumptions were that 25% of the women without the exposure (without *KIR AA* genotype) had persistent hypertension and that the risk was twice among the exposed (with *KIR AA* genotype). A sample size of 127 gave the study 80% power to detect the effect of *KIR* genotype on persistence of hypertension, including 8% loss to follow up. 

The clinical management of all the participants was according to Mulago Hospital’s standard protocols. All women with diastolic blood pressures of 110 mmHg or more were diagnosed as severe pre-eclampsia and were given magnesium sulphate therapy as follows: a loading dose of magnesium sulphate of 14 g was given (4 g intravenously and 10 g intramuscularly), followed by 5 g intramuscularly four hourly for 24 hours. Intravenous hydralazine 5 mg was also given every 30 minutes until the diastolic pressure was 100 mmHg or less. Thereafter, the blood pressure control was achieved using oral nifedipine 10 mg with or without aldomet 250 mg. Those diagnosed as mild pre-eclampsia (diastolic blood pressure of less than 110 mmHg) were given oral nifedipine 10mg with or without aldomet 250 mg or atenolol 50 mg. The decision to deliver the women and mode of delivery plus subsequent care was also according the hospital’s standard protocols. For the first week all the pre-eclamptic women were left on antihypertensive drugs. In subsequent reviews the doses of the antihypertensive drugs were changed or the treatment was stopped altogether, depending on the blood pressure measurements. Treatment was stopped in those women whose blood pressure measurements fell below 120/70 mmHg and they were followed up weekly for three weeks. Those whose blood pressure measurements remained at or below 120/70 mmHg during this period were told to return or call in case of complaints like headache, but if all remained well they were told to return at 3 months post delivery for the final evaluation.On the other hand, the women whose blood pressure measurements fluctuated between 120/70 and 140/90 mmHg continued with fortnightly reviews and if the blood pressure measurements went above 140/90 mmHg they were put back on antihypertensive treatment. Fortnightly follow up reviews continued until the measurements fell below 120/70 mmHg or until 3 months post delivery, whichever came first. 

### Data analysis

Data was entered into Access databases and the datasets were later imported into and analyzed using STATA version 12. First, we investigated the normality of the data for the continuous variables graphically using histograms and in addition we used the Shapiro-Wilk tests for normality. Then baseline characteristics were compared between those who had persistent hypertension and those who resolved within three months after delivery. Categorical data was compared using the chi-square test while for numerical data the Student’s t-test. To assess risk factors for persistence of hypertension, univariable analysis was performed to compute risk ratios at the 95% significance level. To assess independent contribution of these risk factors to persistent hypertension, multivariate analysis was performed, where all independent variables with a p-value of less than 0.2 at univariate analysis were considered for multivariable logistic regression. Associations with p values less than 0.05 were considered statistically significant.

## Results

A total of 200 were recruited into this prospective cohort study. All the eligible women had reliable mobile phone contacts but 4 were excluded from the study because they intended to reside beyond a radius of 20 Km after delivery. Of the 200 women recruited, 12 failed to attend for post-natal review leaving data for 188 women for analysis 2 women had no *KIR* genotyping results. Out of the 188 women, 64 (34%) had persistent hypertension at three months post delivery. 

All continuous variables (women’s age, blood pressure, birth weight, gestational age) were normally distributed, with p-values of less than 0.001 for all variables from the Shapiro-Wilk tests. Table 1 compares the demographic and clinical characteristics between women with and without persistent hypertension. Women who had persistent hypertension were older than those who became normotensive. The gestational age at delivery and birth weight were lower among women who had persistent hypertension. More women in this group were multiparous and had HIV infection. None of the women reported ever smoking and all women had conceived naturally.

**Table 1:** Comparison of characteristics of women with and without persistent hypertension at 3 months after delivery.

		**Persistent hypertension**	**Normal blood pressure**	
**Characteristics**	**Categories/measurements**	**N=64**	**N=124**	**P-value**
		**n (%)**	**n (%)**	
**Women’s age**	mean years ± SD	27.3 ± 6.0	24.0 ± 4.3	< 0.001
**Gestational age at delivery**	mean ± SD in weeks	35.6 ± 4.0	37.6 ± 3.4	0.001
**Baby’s birth weight**	mean ± SD in Kg	2.3 ± 0.87	2.7 ± 0.83	0.011
**Parity**	Primigravidae	17 (26.6)	72 (58.1)	< 0.001
	Multiparous	47 (73.4)	52 (41.9)	
**Women’s ethnic group**	Bantu	61 (95.0)	109 (87.9)	
	Luo	1 (1.6)	6 (4.8)	0.304
	Nilohamites	0	4 (3.2)	
	Others	2 (3.1)	5 (4.0)	
**Past miscarriage**	No	52 (81.2)	113 (91.1)	0.050
	Yes	12 (18.7)	11 (8.9)	
**Mode of delivery**	Spontaneous vaginal	17 (26.6)	14 (11.3)	
	Induced vaginal	47 (73.4)	30 (24.2)	0.292
	Caesarean	35 (54.9)	80 (64.5)	
**Women’s *KIR* genotype**	*KIR AA*	25 (39.1)	43 (35.2)	0.608
	Not *KIR AA*	39 (60.9)	79 (64.7)	
**HIV status**	Negative	59 (92.2)	123 (99.2)	0.018
	Positive	5 (7.8)	1 (0.8)	

Table 2 shows the univariable analysis for factors associated with persistent hypertension at three months after delivery. The factors that were significantly associated with persistent hypertension were the women’s age, parity, gestational age at delivery, HIV status and severity of pre-eclampsia. On the other hand, delivery at term (that is, 38 week of gestation or more) was associated with resolution of pre-eclampsia at three months after delivery. Maternal *KIR* genotype was not associated with persistent hypertension.

**Table 2:** Univariable analysis for factors associated with persistent hypertension at three months after delivery.

**Characteristics**	**Categories**	**Crude risk ratios**	**95% confidence intervals**	**P-value**
**Women’s age (years)**	13-19	1.00		
	20-24	0.96	0.33 - 2.79	0.942
	25-29	1.50	0.51 - 4.40	0.460
	30-34	4.43	1.30 - 15.09	0.017
	35 and above	28.5	2.97 - 273.31	0.004
**Parity**	Multiparous	1.00		
	Primigravidae	0.26	0.13 - 0.50	< 0.001
**Women’s *KIR* genotype**	Not *KIR AA*	1.00		
	*KIR AA*	1.18	0.63 - 2.20	0.608
**Gestational age (weeks)**	24-34	1.00		
	35-37	0.52	0.23 -1.18	0.120
	38 and above	0.32	0.15 - 0.67	0.003
**HIV status**	Negative	1.00		
	Positive	10.42	1.19 - 91.23	0.034
**Urine protein level at recruitment**	2+	1.00		
	3+	0.91	0.41- 2.01	0.815
	4+	1.76	0.84 - 3.69	0.132
**Previous hypertension in pregnancy**	No	1.00		
	Yes	0.79	0.30 - 2.08	0.633
	No prior pregnancy	0.25	0.12 - 0.49	< 0.001
**Severity of pre-eclampsia**	Mild	1.00		
	Severe	3.85	1.69 - 8.80	0.001

We then analysed for factors independently associated with persistent hypertension and the results are shown in Table 3. Women’s age (especially among those aged 35 years or more) and multiparity were statistically significant in predicting persistent hypertension. On the contrary, delivery at term was significantly not associated with persistent hypertension. 

**Table 3:** Predictors of persistent hypertension at three months after delivery.

**Characteristics**	**Categories**	**adjusted risk ratios**	**95% confidence intervals**	**P-value**
**Women’s age (years)**	13-19	1.00		
	20-24	0.86	0.28 - 2.60	0.787
	25-29	1.04	0.32 - 3.32	0.946
	30-34	2.37	0.63 - 9.00	0.202
	35 and above	15.30	1.47 - 159.04	**0.022**
**Parity**	Multiparous	1.00		
	Primigravidae	0.46	0.21 - 0.99	**0.048**
**Women’s *KIR* genotype**	Not *KIR AA*	1.00		
	*KIR AA*	1.25	0.62 - 2.53	0.527
**Gestational age (weeks)**	24-34	1.00		
	35-37	0.50	0.20 -1.25	0.138
	38 and above	0.39	0.17 - 0.90	**0.028**

In this cohort we defined a group of high risk women for persistent hypertension: multiparous, 25 years of age or more and with early onset pre-eclampsia (delivered at gestational age of 24-34 weeks). From the analysis 21 of the 188 women (11.2%) were high risk and a higher proportion of these, 12 (57.1%), had persistent hypertension compared to 52 (31.1%) of the others. This difference was statistically significant (p-value =0.018). 

## Discussion

In this study we assessed the incidence and factors associated with persistent hypertension after delivery among women with pre-eclampsia/eclampsia who delivered in Mulago Hospital. The incidence of persistent hypertension three months after delivery was 34% (64/188). Women’s age (especially 35 years and above), gestational age at delivery and parity of the women were the factors predictive of persistent hypertension in this cohort. 

The incidence of persistent hypertension we found (34%) is higher than that found among similar women in Mulago Hospital (28%) in an earlier study in which the follow up period after delivery was six weeks [24]. Our slightly higher figure may be due to the fact that this earlier study, unlike ours, excluded women with a history of hypertension in previous pregnancies. These women have been found to be more at risk of later hypertension and cardiovascular disease in some studies (although not in our adjusted analysis) [20,21]. Despite differences in the recruitment criteria both these studies show that persistence of hypertension after pre-eclampsia is common in our institution. This trend is likely to be the same in the rest of sub-Saharan Africa but poor medical records makes it difficult to appreciate the full extent of the problem. The aetiology of the persistent hypertension or later diseases following pre-eclampsia is unclear [11] but could arise as a consequence of pre-eclampsia or because of shared risk factors with pre-eclampsia. In support of the former, markers of endothelial activation persist up to two years after delivery in women who have been affected by HELLP syndrome, a severe form of pre-eclampsia [37]. Thus, pre-eclampsia is likely to contribute significantly to the increasing burden of non-communicable diseases in Africa [38,39] because the risk of pre-eclampsia is also high [40].

We also found that the increased risk of persistent hypertension was highest in women aged 35 years and above. Higher maternal age was associated with persistent hypertension and later cardiovascular disease in other studies [20,24]. Multiparous women were also at increased risk of persistent hypertension. The risk of essential hypertension increases with age [41] and therefore persistent hypertension after pre-eclampsia among older women could be due to previously undiagnosed chronic hypertension. We acknowledge this as a limitation of our study. The fact that multiparous women are also at increased risk further supports this possibility since these are expected to be older. In our setting with limited records documenting past medical history and few routine health checks, there are difficulties in diagnosing hypertension in our population. A recent study of over 150,000 participants showed that it is uncommon for people in low-income settings like ours to be aware that they have hypertension compared to populations with better economic status [42]. Nonetheless we are unlikely to have major biases in selection of participants and our data may well reflect the true prevalence of persistent hypertension in our setting. Our cohort consists of only women, known to have better health seeking behaviour than men and more likely to be aware if they had been diagnosed with hypertension [43]. In addition, women have more opportunities to attend clinics such as the antenatal, post natal and family planning clinics where BP measurements are made. Self reported diagnoses do show substantial agreement with vital statistics and provide valuable information where medical records are scarce [44,45]. In addition the urban location and catchment area of Mulago Hospital with better access to health care [42] means that the women attended to are more likely to be aware they have hypertension, further minimising the likelihood of missing those with prior hypertension at recruitment. Thus, though the possibility of superimposed pre-eclampsia is a limitation of our study, our findings make a strong case for closely monitoring all women who have delivered following pre-eclamptic pregnancies. The postpartum follow up provides an opportunity to catch those women with chronic hypertension regardless of whether they had it before pregnancy or not and therefore minimize the long term complications of the disease.

 When women with pre-eclampsia deliver at term, there was a significantly reduced risk of persistent hypertension in our study. Other studies have also found that it is particularly early onset pre-eclampsia (before 34 weeks of gestation) that is a risk factor for later cardiovascular disease [20,21,23,46]. Early onset pre-eclampsia is usually more severe and more likely to recur [20,21,47]. The severe endothelial disorder that gives rise to the systemic disorder of pre-eclampsia could cause a permanent change in the vasculature predisposing women to hypertension and cardiovascular disease [11]. Alternatively, women with early onset pre-eclampsia might share genetic, or metabolic features that give a susceptibility predisposing them to both pre-eclampsia and cardiovascular disease since some of the risk factors are shared. Pre-eclampsia is typically a disease of primigravidae and when multiparous women develop the disorder the pre-eclampsia is likely to be recurrent [30,48,49]. This points to a possibility that genetic susceptibility could be responsible for the higher risk of both pre-eclampsia and persistent hypertension among the multiparous women, and therefore it is not just a case of superimposed pre-eclampsia. Many risk factors for pre-eclampsia such as a previous history of pre-eclampsia, obesity, diabetes mellitus, chronic renal disease, are known to be the same as those for development of later diseases [10,30] and this makes the idea of a similar underlying aetiology reasonable. Our preliminary findings show an even higher risk group of women for persistent hypertension and these are the multiparous, at 25 years of age or more and with early onset pre-eclampsia. Women who fall in this high risk group should be followed up even more closely than the others and there is need to focus further research on this group.

NK cells through their Killer Immunoglobulin-like receptors (KIR) are believed to play a key role in placentation [50,51] and pre-eclampsia is widely believed to arise primarily as a consequence of defective placentation. Particular *KIR* genes have been implicated in development of pre-eclampsia in some studies [28,51,52] and we hypothesized that these genes may also be associated with resolution of pre-eclampsia (persistence of hypertension). This was premised on the fact that the hypoxia that results from abnormal placentation may cause release of factors that lead to endothelial dysfunction and subsequent multisystem organ dysfunction [29]. The resultant organ dysfunction could lead to persistence of hypertension depending on the severity of the endothelial dysfunction which could be partly dependent on the woman’s her *KIR* genotype and therefore NK cell activity. In this study we found no association between maternal *KIR* genotype and persistent hypertension. This could be explained by the fact pre-eclampsia is a disease of multiple aetiologies and placentation defects, where *KIR* and *HLA-C* genes may play a critical role [51,53,54], are just one possibility. 

In this study there are several other limitations. All women received antihypertensive therapy before and after delivery but we did not, however, collect the data detailing the therapy given such as; the number of drugs, doses, duration of treatment before delivery. This would have been informative since antihypertensive therapy modifies the progression of pre-eclampsia [55,56] and possibly resolution of the disease as well. Detailed data on the use of magnesium sulphate was also not available though all women with severe pre-eclampsia received magnesium sulphate therapy before delivery. This would be important information since magnesium sulphate therapy is known to modify the progression of pre-eclampsia as it causes peripheral vasodilatation which leads to reduction in blood pressure [57]. Participants’ data for biochemical tests such as serum creatinine and uric acid levels was not available and yet these are known to be predictive of persistent hypertension [24]. This study has however shown that even without laboratory tests there are other patient characteristics that can be used to predict persistence of hypertension in a resource limited setting. 

There are still few studies on the persistence of hypertension following pre-eclampsia, with most published literature describing risk of later disease long after delivery [10,12,58]. In addition very few of these studies have been conducted in Africa despite the high morbidity and mortality due to pre-eclampsia that has been observed there [2,59]. This makes comparisons with our study difficult since the aetiology of persistent hypertension may not necessarily be similar to that of later hypertension or cardiovascular disease and may differ in different settings. Furthermore, because pre-eclampsia is a heterogeneous disorder its effects after delivery are also likely to be as such. Therefore more follow up studies of women affected by pre-eclampsia, especially among women of African ancestry where pre-eclampsia is prevalent and also appears more severe [30-34], need to be done in different settings. This way, the risk factors for both persistence of hypertension and later disease will be more quickly unravelled. 

## Conclusion

The proportion of women with pre-eclampsia/eclampsia at risk of persistent hypertension three months after delivery in our setting is high, with nearly one of every three mothers remaining hypertensive. This trend could be similar in other African countries, contributing to the increasing burden of non-communicable diseases that has been observed [38,39]. Maternal age, gestation age at delivery and parity are predictors of persistent hypertension at three months after delivery for women with pre-eclampsia and eclampsia. Follow up of mothers who have had pre-eclampsia is very important so that diagnosis and management of chronic hypertension is made early before complications develop.
